# Bioanalytical Application of the Total-Reflection X-Ray Fluorescence Spectrometry

**DOI:** 10.3390/ijms26031049

**Published:** 2025-01-26

**Authors:** Ramón Fernández-Ruiz

**Affiliations:** Servicio Interdepartamental de Investigación, Laboratorio de XRF (TXRF/GIXRF/μXRF/PSD-LD), Universidad Autónoma de Madrid, Ciudad Universitaria de Cantoblanco, 28049 Madrid, Spain; ramon.fernandez@uam.es

**Keywords:** TXRF, microanalysis, atomic analyses, trace metals, biomedicine, biomaterial, pharmacology, cancer, diseases, diagnostic

## Abstract

This paper briefly overviews the application of total-reflection X-ray fluorescence (TXRF) spectrometry in the biosciences, focusing on key bioanalytical applications. It seeks to review and update the current state of TXRF’s use in biomedical, biochemical, and pharmacological research. The review highlights relevant works in the field, summarising past achievements and incorporating the latest developments. The goal is to demonstrate how the analytical application of TXRF spectrometry in this area has evolved and what its role is in analysing trace elements and other biomolecules in diverse biological samples and diseases. Physical foundations to understand its analytical power and its comparison with related analytical techniques are presented to gain objective knowledge of the benefits, limitations, and drawbacks that TXRF spectrometry can offer.

## 1. Introduction

Elemental analysis plays a crucial role in biomedicine, biochemistry, and pharmacology by providing essential insights into the composition of biological samples. By accurately determining the elemental composition of biological molecules, tissues, and drugs, researchers can gain a deeper understanding of their properties and functions at a molecular level. This analytical technique enables scientists to identify critical elements in biological systems, uncover trace elements that may have significant biological effects, and track changes in elemental concentrations associated with various diseases or drug treatments. Ultimately, elemental analysis helps advance our knowledge of the intricate relationships between elemental components and biological processes, paving the way for developing novel therapeutic strategies and diagnostic tools in these critical scientific disciplines [1]. In this sense, TXRF is an alternative and not well-known analytical technique that can contribute to advancing research in this area of knowledge. One of the main objectives of this paper is to try to show these analytical potentialities and provide a brief background of relevant applications In the biomedical, biotechnological, and biochemical interest areas. TXRF is presented and compared with related techniques, aiming to give researchers an objective vision of the potential help that TXRF can offer to their research.

Some recently published papers concisely overview TXRF spectrometry’s relevance and utility in bioanalytical research [2,3]. They aimed to summarise this field’s essential findings and trends, including pharmacological applications. The papers delve into the specific applications of TXRF spectrometry within bioanalytical sciences and serve as a reference for this work. The idea is to cover the TXRF use in trace element analysis, quantification of biological samples, and its role in studying various biological matrices. This paper discusses the more outstanding and recent advancements in TXRF spectrometry related to its application in the bioanalytical field. Additionally, this work addresses challenges associated with TXRF spectrometry in bioanalysis, such as sample matrix effects or instrument limitations, suggesting future directions for research or improvements in the technique.

TXRF spectrometry is a highly sensitive analytical technique that quantitatively determines elements in small sample quantities. One of its main advantages is its ability to analyse minimal sample amounts, achieving detection limits in the range of picograms or concentrations as low as a few ppb (μg/L). Due to its microanalytical nature, TXRF is especially useful for studying samples with limited amounts. This fact makes it possible to perform qualitative and mass ratio analyses of the elements in a sample of only a few hundred nanograms. These abilities are of great interest in researching metal traces in biological, biomedical, or pharmacological fields because these application fields require the rapid, precise, and accurate characterisation of elemental fingerprints at trace and ultra-trace levels. So, this paper shows the application of its analytical abilities for very diverse kinds of biomatrices, i.e., microtomic sections, human fluids such as blood, serum, amniotic fluid, cerebrospinal fluid, oral fluid, urine, seminal fluid, and metalloproteins or cytosol pellets. Also, for cancer research, particularly for evaluating antitumoural compounds’ action and researching tumoural/cancerous tissues.

Reinhold Klockenkämper and Alex Von Bohlen have published a second edition of their unique monographic book on TXRF [4]. Nowadays, this book represents the essential starting point for beginners and experts to learn and practice TXRF spectrometry. This concise review provides an overview of the physical principles of TXRF, highlighting its analytical advantages and limitations with improved clarity. Additionally, it presents a selective summary of the most notable applications of TXRF spectrometry in bioanalysis.

### 1.1. Basis of the TXRF Technique

In 1923, Compton discovered the total reflection of the X-ray [5]. He demonstrated that the X-ray reflectivity of a flat material rises below a specific angle reliant on the material serving as the reflector and the energy of the incident X-ray. Among the TXRF community, this angle is known as the critical angle. For a Mo Kα source with a quartz reflector, the critical angle is approximately 0.1 degrees. It was not until 1971 that Yoneda and Horiuchi proposed investigating the excitation–emission X-ray fluorescence of atoms deposited on a reflector using total reflection geometry [6]. This method leverages the total reflection effect to enhance the excitation region through dual excitation of the sample by both the incident and the totally reflected beams. This groundbreaking concept led to several innovative studies during the 1970s and early 1980s [7,8].

**Figure 1:**
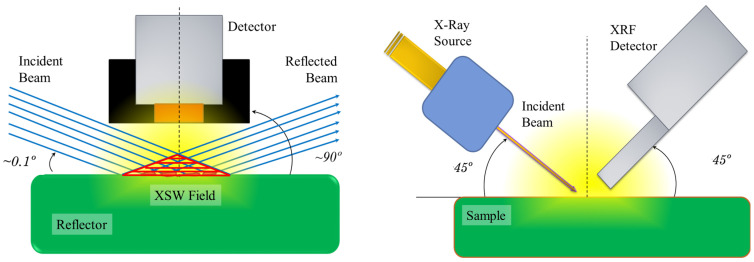
Vertical section of the geometry over a TXRF reflector (**left**) compared with conventional XRF geometry (**right**).

It Is Interesting to clarify the particular reason for the high sensitivity of TXRF spectrometry to understand its potential applicability. TXRF is an X-ray spectrometric technique derived from classical energy-dispersive X-ray fluorescence (EDXRF). The main difference is basically the geometrical arrangement of the source-sample detector. So, working in the total reflection condition implies the interaction of an incident beam of an monochromatic electromagnetic wave with the equivalent reflected beam. Then, both beams interact in a region of the space over the sample support reflector. The constructive interference of both electromagnetic waves generates a field of X-ray standing waves (XSW) over the surface of the sample carrier, where the sample is introduced for analysis. This XSW resonator region is the key to the outstanding analytical properties of the TXRF technique. Figure 1, left, shows the geometrical configuration of TXRF and the XSW field region compared with conventional XRF geometry. The perspective is a vertical section of the parallelepiped region. Therefore, the XSW field region is visualised as a triangle. Figure 1, right, shows the conventional geometrical basic XRF arrangement where the more significant differences are the incident and detection angles. The incidence angle varies from 0 to 45° for TXRF and XRF, respectively. The detection angle varies from 90° to 45° for TXRF and XRF, respectively.

Therefore, the TXRF is a microanalytical tool that can analyse minimal sample quantities. Thus, even a few micrograms of the sample can be analysed with relative ease, as the detection limits of the mass sample of the elements are quantified in the order of picograms deposited over the reflector surface into the XSW region. TXRF microanalytical capability is a powerful tool for analysing pharmacological or bio-samples since these samples are usually available in small quantities.

Today, TXRF instrument costs are competitive with traditional flame or plasma instruments, AAS, or ICPS, making them accessible across scientific and industrial sectors requiring metal trace analyses. TXRF boasts significantly lower use and maintenance expenses than flame or plasma instruments, requiring only a modest electric connection alongside standard lab equipment to achieve high-quality results at ppb levels. Biennial conferences have propelled the development of TXRF spectrometry focused on TXRF and related methodologies globally. Notable international conferences on TXRF have been held in various locations, including Geesthacht, Germany (1986, 1992); Dortmund, Germany (1988, 1996, 2011); Vienna, Austria (1990, 2000); Tsukuba, Japan (1994); Eindhoven, The Netherlands (1996); Austin, TX, USA (1998); Madeira, Portugal (2002); Awaji Island, Japan (2003); Budapest, Hungary (2005); Trento, Italy (2007); Gothenburg, Sweden (2009); Osaka, Japan (2013); Denver, CO, USA (2015); Brescia, Italy (2017); Girona, Spain (2019); and Clausthal, Germany (2023). TXRF has demonstrated its versatility through successful applications in challenging materials and diverse scientific fields, including medicine [9], environmental sciences [10], archaeology [11], materials physics [12], electronics [13], art [14], nanotechnology [15], biology [16], and more [4]. Despite significant progress, there is untapped potential for TXRF applications, as shown by a gradual rise in scientific publications related to TXRF starting from the commercialisation of the first instruments in 1985 by Rich & Seifert in Germany. The TXRF analytical technology has gained recognition within the international scientific community over the years, signifying a promising future for its continued development and applications.

**Figure 2:**
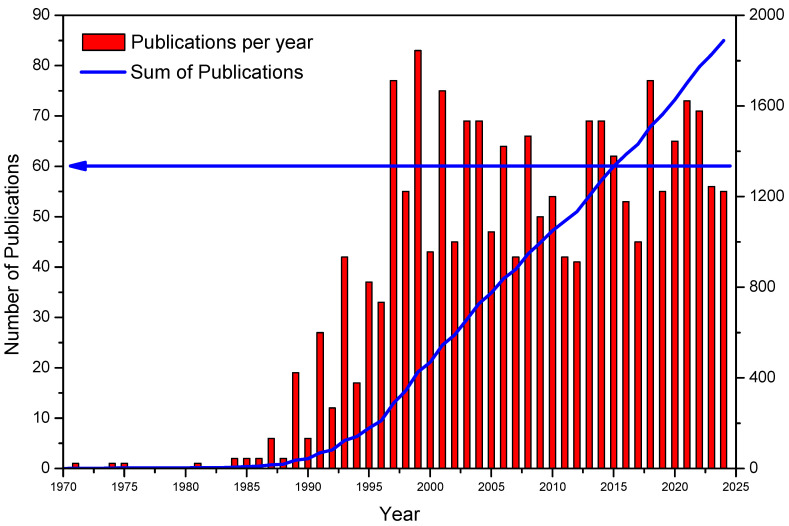
Papers published by year (bars) and their accumulated sum (line), where “TXRF” has been used as a keyword in the Web of Science Clarivate^®^ database 2024.

The present TXRF database contains about 1900 international scientific publications, indicating numerous scientific fields where TXRF can yet showcase its capabilities. From 1985 to 2000, scientific productivity increased steadily, stabilising at an average value of 60 international publications annually. Figure 2 illustrates this trend, showing periodic biannual increases that align with the years following international TXRF conferences. As a result, contributions presented at these conferences are frequently compiled and published in special issues of *Spectrochimica Acta Part B: Atomic Spectroscopy*. Along with the TXRF monograph by Reinhold Klockenkämper and Alex von Bohlen [4], these publications serve as foundational technical resources in the field.

### 1.2. Physical Background

**Figure 3:**
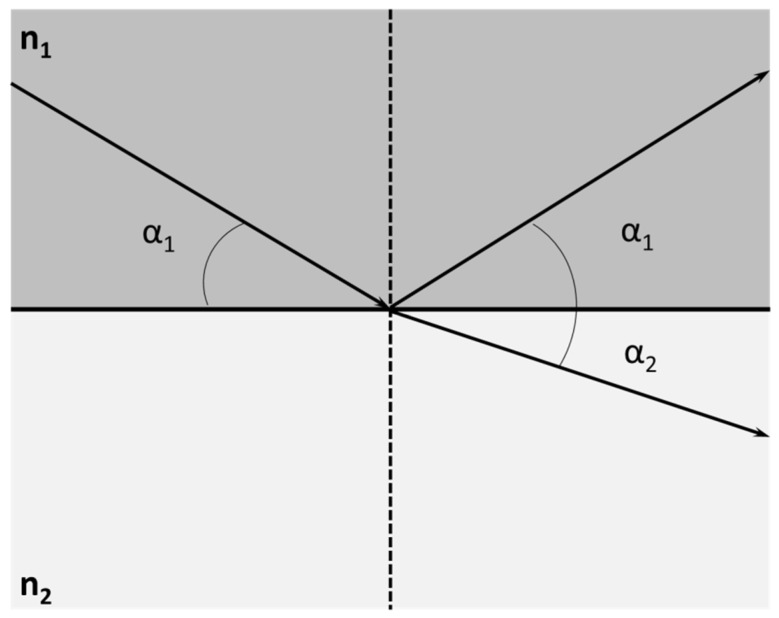
Reflection and refraction of an X-ray beam crossing two mediums with refractive index *n*_2_ < *n*_1_.

An X-ray beam travels in a straight line through a homogeneous medium. However, if the beam encounters a boundary with a new medium, its behaviour changes. Similar to visible light, part of the incident X-ray beam is reflected back into the original medium. At the same time, the rest is refracted into the second medium (as shown in Figure 3). The relationship between the angle of incidence α_1_ and the angle of refraction α_2_ is described by Snell’s law:

(1) $$n_{1}\cos\alpha_{1}=n_{2}\cos\alpha_{2}$$

where *n*_1_ and *n*_2_ are the refractive indices of media 1 and 2, respectively. For X-rays, any medium is optically less dense than a vacuum, and any solid is optically less dense than air. This fact implies that the refractive index of a solid is lower than that of air, i.e., *n_solid_* < *n_air_*. If the angle α_2_ is zero, the refracted beam will emerge tangentially to the interface. Therefore, there must be a critical angle for the incident beam, α_1_ = α_c_, where the refracted beam acquires an angle α_2_ = 0.

According to Snell’s law, and considering that *n_air_*~1,

(2) $$\cos\alpha_{c}=n_{solid}$$

Moreover, the refractive index of the solid reflector has the complex form

(3) $$n_{solid}=1-\delta -i\beta$$

where *i* is the imaginary unit and the parameters *δ* and *β* are the scattering and absorption parameters of the sample-support material, respectively. From Equations (2) and (3) we can estimate the value of the critical angle of any material in the form

(4) $$\alpha_{c}\approx \sqrt{2\delta }\approx \frac{1.65}{E}\sqrt{\frac{Z_{m}}{A_{m}}}\rho ,$$

where *E* is the incident photon energy (keV), *Z_m_* is the atomic number, *A_m_* is the atomic weight (g/mol), and *ρ* is the density of the material (g/cm^3^) [4].

**Figure 4:**
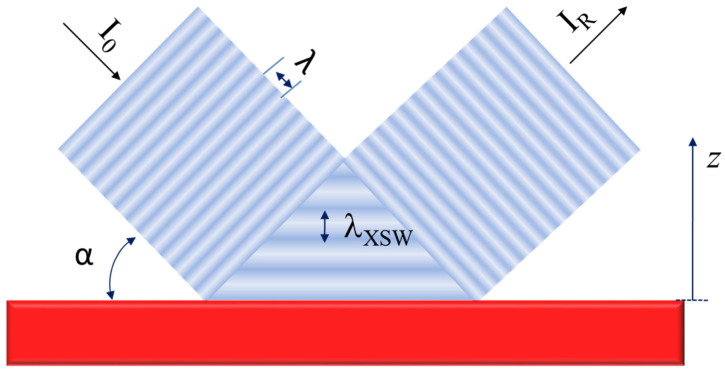
Cross-section of the X-ray standing waves field generated over a reflector’s surface below its critical angle. *I*_0_ and *I_R_* represent incident and reflected intensities. *λ* and λ_XSW_ describe the wavelength of the incident electromagnetic wave and the wavelength of the XRS field, respectively.

For incidence angles below the critical angle α_c_, Snell’s law does not yield real values for the refraction angle α_2_. In this regime, the X-ray beam penetrates only a few nanometres into the second medium, and the interface acts as a nearly perfect mirror, fully reflecting the incident beam back into the first medium. Consequently, the incident and reflected beams interfere constructively at angles below the critical angle, producing a field of X-ray standing waves (XSW) at the interface, as illustrated in Figure 4.

**Figure 5:**
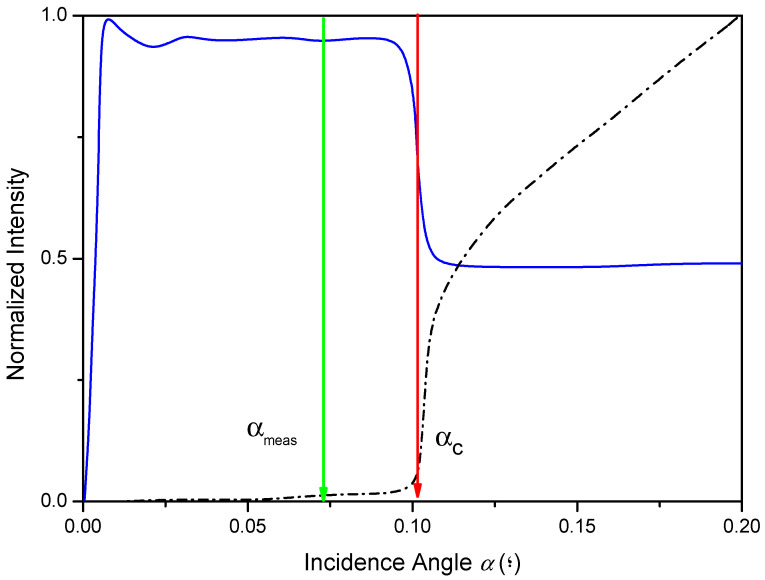
The blue line displays the normalised Fe Kα fluorescence signal of a sample deposited as a film of 500 nm (Equation (5)) around the critical angle. The black line shows the normalised background signal of a silicon reflector (Equation (6)) around the critical angle vicinity. The red arrow marks the critical angle α_c_ for the silicon sample support. The green arrow marks the angle of measurements α_meas_ assuring the TXRF condition. Excitation source of Mo Kα (17.4 keV).

The phenomenon of constructive interference generated in the XSW field implies that the intensity inside the interference region is amplified. The amplitude of the XSW field can be up to twice the amplitude of the incident electromagnetic waves. Still, as the intensity of the XSW field is proportional to the square of the amplitude, its value can be up to four times higher than the incident beam. So, this amplified XSW field is confined to a limited region of the space where the incident and reflected monochromatic electromagnetic waves interact. On average, the intensity amplification is around twice that of conventional XRF. If a sample is deposited into the XSW region, it is excited twice. Another way to understand this amplification effect is by considering that the atoms are excited simultaneously by the incident and the totally reflected beam. The stationary wave field’s cross-section is triangular, and I like to call it a “magic triangle” or “resonator”. The general equation for the intensity distribution, *I*(*α*,*z*), in the X-ray standing wave (XSW) field describes how the intensity depends on the incident angle, α, and the position, z, above the reflecting surface [4]. This theoretical framework is crucial for understanding the angular and spatial characteristics of XSW fields, enabling precise analysis of atomic-scale structures near surfaces and interfaces. Equation (5) gives the shape of the XSW intensity distribution as

(5) $$I\left(\alpha ,z\right)=I_{0}\left[1+R\left(\alpha \right)+2\sqrt{R\left(\alpha \right)}\cos\left(\frac{2\pi z}{\lambda_{XSW}}-\phi \left(\alpha \right)\right)\right],$$

where *I*_0_ is the intensity of the primary beam, and the cosine argument is the phase difference between incident and reflected waves, broken down into two components, a spatial 2*πz/λ_XSW_* and a phase shift *ϕ(α)*. Finally, *R(α)* is the reflectivity of the material used as a reflector, or sample carrier, for each angle of incidence α. Then, if a sample is introduced inside the XSW field, the atoms will be excited by this X-ray intensity distribution. Consequently, the fluorescence emissions intensity will be proportional to the intensity *I*(*α*,*z*) of the XSW field. Now, assuming that the contribution to the spectral background of the sample carrier is infinitely thick, optically flat, and homogeneous, the intensity of the evanescent wave field that penetrates the reflector is given by the equation

(6) $$I\left(\alpha ,z\right)=I_{0}\left[1+R\left(\alpha \right)+2\sqrt{R\left(\alpha \right)}\cos\left(\phi \left(\alpha \right)\right)\right]\exp\left(-\frac{z}{z_{e}}\right),$$

where the depth of penetration *z_e_* is defined as the depth at which the X-ray beam penetrates in the medium, such as its intensity being reduced by a factor of 1/*e*. Note that the evanescent wave’s penetration is only a few nanometres and remains practically constant below the critical angle in the total reflection condition. Figure 5 shows the intensity signal of X-ray fluorescence (Fe Kα) associated with a sample of Fe deposited with a typical thickness of 500 nm on a Si reflector (blue), next to the Si Kα X-ray fluorescence signal (black) of the Si reflector acting as sample support. Both curves are displayed versus the angle of incidence of the X-ray source beam.

Evaluating Figure 5, below the critical angle of the reflector *α_c_*, the TXRF measurement region, the Fe sample fluorescence emission intensity (blue) is approximately twice that for angles higher than α_c_, the conventional XRF measurement region.

Simultaneously, the Si Kα background intensity (black) below the critical angle α_c_ is extremely low and decreases quickly because the intensity of the incident X-ray beam is practically reflected. The remaining intensity penetrates only a few nanometres in the reflector material. For angles above the critical angle α_c_, the contribution of the spectral background increases quickly and linearly. The spectral background decreases by a factor of around 500 from work down α_c_ in the TXRF region or above α_c_ in the conventional XRF region.

This combination of phenomena gives the TXRF its analytical power. Combining this reduction in the background with factor two associated with amplifying the sample fluorescence signal below the critical angle, the signal-to-noise ratio obtained in the TXRF condition over conventional XRF is up to three orders of magnitude higher. Therefore, the resulting increase in sensitivity allows the TXRF to detect masses deposited on the reflector’s surface of only a few picograms. In this way, if the measurement angle α_meas_ is fixed around 70% of the critical angle α_c_ (see Figure 5), the TXRF condition is assured. The main consequence of this inherent characteristic of the TXRF is that the simple sample deposition over the surface of an adequate reflector allows the analysis of the present element with the best signal-to-noise ratio and with lower detection limits than the X-ray spectrometry can get today. From a physical perspective, this is the main differentiating factor of TXRF over any other conventional XRF spectrometries.

### 1.3. Analytical Background

**Figure 6:**
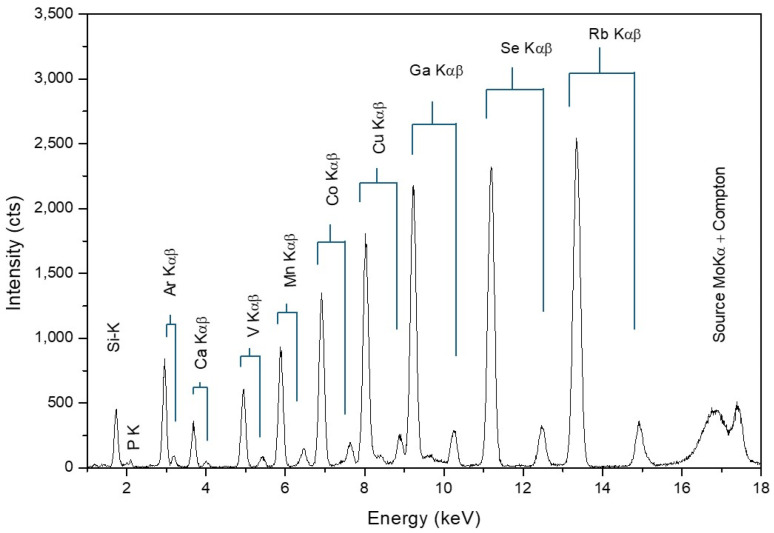
A TXRF spectrum of a multielement standard solution, with each element deposited at a mass of 10 nanograms, was recorded using an incident Mo Kα X-ray beam and an acquisition time of 100 s. The spectrum displays the couple of K-lines for each deposited element (Kα the highest peak and Kβ the lower peak), the Si signal originating from the quartz reflector, and the Compton and Rayleigh signals from the X-ray source.

TXRF is classified as a “micro-analytical” technique because it can quantify small sample amounts, typically ranging from 0.1 to 10 micrograms, carefully placed on a reflector for analysis. The deposition thickness is typically down to 10 μm, varying based on the kind of matrix. These conditions enable the assumption of an infinitely thin film model with negligible impacts from absorption and secondary excitation. These facts lead to a significant advantage in TXRF, where matrix effects can generally be disregarded during quantification. Unlike conventional XRF techniques, this unique aspect of TXRF simplifies sensitivity assessments. By minimising and bypassing the matrix effect, TXRF achieves relative sensitivities with a universal character, independent of the analysed matrix. Determining the relative sensitivities of a TXRF instrument is a straightforward process. Figure 6 illustrates a TXRF spectrum obtained from the deposition of 10 microlitres of a multielement standard solution onto a quartz reflector, with each element present at a concentration of 1 ppm (ng/μL). Consequently, the net mass of each deposited element was 10 nanograms. Using certified standards, where the absolute mass of a deposited element is known, the relative sensitivities of each element can be easily calculated. Regarding the elemental range of TXRF, all elements with atomic numbers (Z) greater than 11 can be analysed when an appropriate excitation source is employed.

Conventional TXRF instruments face difficulty evaluating elements with atomic numbers below 12 due to their low-energy fluorescence emissions, quickly absorbed on their path to the detector by air and the detector window. Only samples with high concentrations of Mg or Na can be analysed to evaluate these light elements. To expand the range of detectable lighter elements in TXRF, Peter Wobrauschek and Christina Streli at the AtomInstitut of Vienna have developed significant instrumental enhancements. Their group created a vacuum-operable TXRF chamber called the “WOBISTRAX”, which, when combined with a high count-rate detector (SDD), an ultra-thin window, and a low-energy excitation source like Cr for optimising low-energy transitions or even synchrotron lines, enables the analysis of elements such as B, C, N, O, F, Na, and Mg. This setup achieves detection limits in the femtogram range using TXRF [17,18].

The sample preparation techniques outlined for TXRF analysis involve the versatility of analysing samples in various ways and solving complex analytical problems. Liquid samples are easily analysed after a simple sample deposition over a reflector for a quick qualitative elemental profile. So, selecting a reference element based on its absence in this profile, liquid samples are standardised using a known concentration of the chosen reference element as an internal standard before the analysis by TXRF. On the other hand, solid samples require prior digestion in an acid medium for complete disaggregation. TXRF also offers the option to analyse solid samples after optimising their suspension in a suitable liquid medium, avoiding the challenges associated with chemical acid digestion. This approach ensures accurate quantitation and promotes environmentally friendly analytical practices.

From an analytical perspective, TXRF offers several advantages over conventional quantitative techniques. One of the key strengths of TXRF spectrometry is the stability of relative instrumental sensitivities. Once determined, these sensitivities remain consistent for years and only require recalibration when the detection system or geometry changes are made. Under these conditions, TXRF demonstrates its full potential in atomic chemical analysis through its straightforward approach to understanding, quantifying, and interpreting spectra.

**Figure 7:**
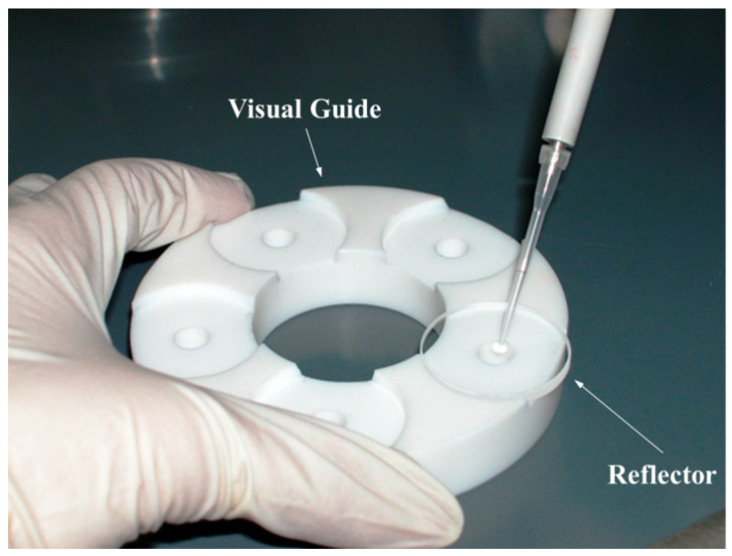
The depositing process of an aliquot of the standardised sample onto a quartz sample carrier using essential tools for TXRF analysis, including a visual guide, a micropipette, and a clean quartz reflector.

In the angular region of total reflection, TXRF simplifies the complex, non-linear matrix effect, which is a significant challenge in conventional XRF quantification. Instead, TXRF achieves a simple and linear relationship for elemental analysis [4]. Consequently, the master equation for TXRF quantification is based on this simple linear relationship:

(7) $$C_{x}=C_{IS}\frac{N_{x}}{N_{IS}}\frac{S_{IS}}{S_{x}},$$

where *C_x_* and *C_IS_* are the concentrations of element *x* and the internal standard, *IS*, *N_x_*, and *N_IS_* are their net intensities, and *S_x_* and *S_IS_* are their sensitivities, respectively. As Equation (7) shows, the internal standard is the most suitable quantification method for the TXRF technique. This method involves adding an aliquot of a known concentration of a single-element standard that is absent in the sample being analysed. Depositing a small portion of this standardised solution onto a clean reflector (see Figure 7) is sufficient to quantify all the elements present in the sample solution.

The sample volume deposited on the reflector can be adjusted within methodical constraints [19] to optimise the counting ratio during spectral acquisition in TXRF analysis. This adjustment allows for a measurable concentration range spanning from a few parts per billion (ppb) to thousands of parts per million (ppm), resulting in an analytical concentration range of 10^5^, i.e., five orders of magnitude in concentration, encompassing the diverse element concentrations within the sample under examination.

**Figure 8:**
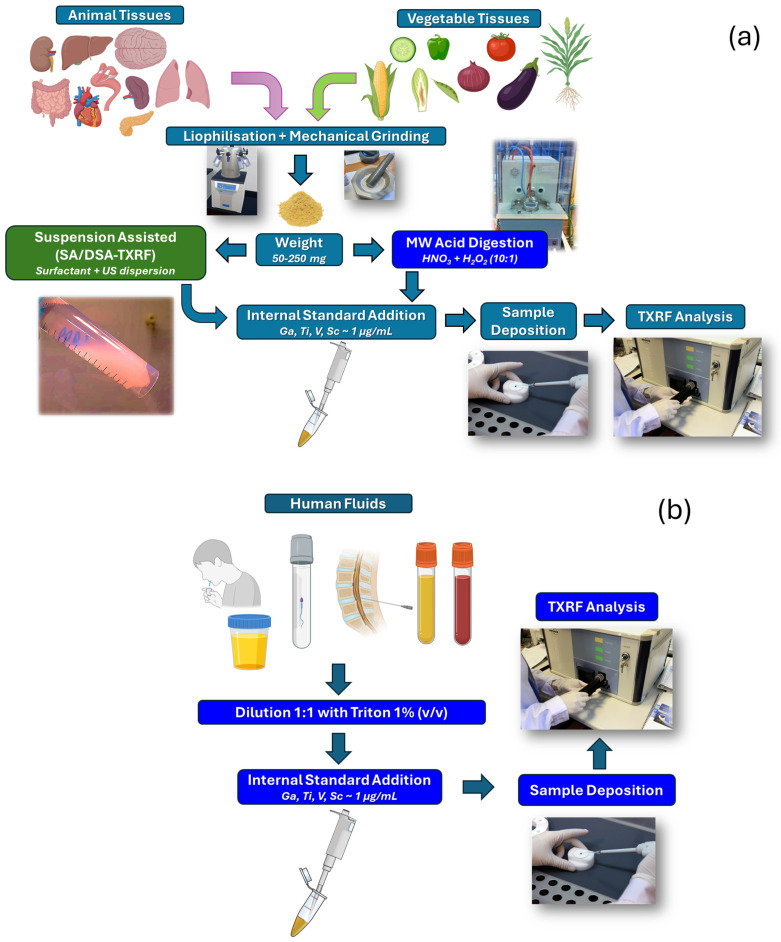
(**a**) General methodologies for the TXRF quantitative analysis of animal or vegetable solid tissues. (**b**) General methodology for the TXRF quantitative animal or vegetable fluids. Some of the images have been generated with BioRender.com (accessed on 17 January 2025).

Considering the sample preparation of biomaterials for the analysis by TXRF, three different approximations can be generally applied: (1) dilution for animal or vegetable liquid samples, (2) lyophilization and mechanical grinding for animal or vegetable solid tissues previous to the acid digestion, or (3) cryogrinding previous to the assisted suspension. After each sample preparation, the analysis methodology is the same, based on the internal standard method described previously. Figure 8a shows the general methodologies for solid tissues, animals, or vegetables, and Figure 8b shows the general methodology for animal or vegetable fluids.

TXRF competes with AAS and ICP-OES due to comparable detection limits (DL) in the range of hundreds of parts per trillion (ppt) to tens of parts per billion (ppb) for liquid samples. Despite this, ICP-MS surpasses these techniques in sensitivity for liquid samples, achieving DLs of around one part per trillion (ppt), making it ideal for ultra-trace analysis. When analysing solid samples using TXRF, the standard practice involves acid digestion, a requirement also in AAS and ICP techniques. Nonetheless, TXRF offers a rapid, straightforward, and direct qualitative and quantitative assessment of element presence in the sample using only a few micrograms of material, without the chemical alterations typical of acid digestion. Furthermore, by employing particle size modulation through different particle-breaking techniques and suspension-assisted methods, TXRF enables the quantitative analysis of solids [20]. This feature underscores TXRF’s significant analytical capabilities and unique microanalytical nature.

TXRF analysis is advantageous due to its capacity for simultaneous qualitative examination of the atomic fingerprint of a material, facilitating monitoring of processes by comparing spectra across phases. This characteristic enables comprehensive analysis of both liquid and solid phases. It aids in tracking lost elements during digestion or induced impurities during preparation. TXRF’s microanalytical nature is beneficial for certain studies but may pose challenges for assessing heterogeneous samples, requiring thorough sample homogenisation and statistical sampling. Unlike flame/plasma techniques, TXRF exhibits low sensitivities for quantifying light elements with atomic numbers lower than 13.

Quantification methods like AAS or ICPS rely on external calibration curves. In contrast, the TXRF technique requires only adding an internal standard element with a known concentration that is absent from the sample. This simplified quantification process significantly reduces both time and costs during analysis.

The “flame/plasma” techniques can be categorised into three tiers based on instrumentation costs. The first tier is atomic absorption spectroscopy (AAS), which includes the graphite chamber and costs approximately EUR 60,000. The second tier includes optical plasma techniques (ICP-OES), which costs around EUR 90,000. The third tier is mass plasma techniques (ICP-MS), with prices of around EUR 160,000.

Similarly, TXRF instruments for analytical applications are available at different price levels. The TXRF WOBISTRAX vacuum chamber from the Atom Institute in Vienna is the most affordable option, priced at approximately EUR 50,000. The next level includes robust spectrometers like the S2 PicoFox, the newer S4 TSTAR from Bruker-nano, and the Horizon spectrometer from GNR, with costs ranging from EUR 70,000 to EUR 100,000. At the highest level are TXRF spectrometers with GIXRF capabilities, such as the NANOHUNTER-II from Rigaku or the enhanced S4 T-STAR from Bruker, priced around EUR 120,000.

Modern TXRF instruments require little more than a standard electrical outlet to operate. In contrast, flame and plasma technologies rely on high-purity gases and other resources, substantially increasing the cost per sample compared to TXRF. This cost disparity becomes even more pronounced when considering the differences in the analytical procedures for sample preparation, standardisation, purity requirements, and the high-purity reagents and materials involved. The simplicity and efficiency of the internal standard method used in TXRF make it faster, easier, and more cost-effective than the external calibration method employed by the flame and plasma techniques.

## 2. Bioanalytical Applications

The total reflection X-ray fluorescence (TXRF) technique has valuable features for bioanalysis applications, such as microanalytical capability, simple sample quantification using an internal standard, low detection limits achieved in the parts per billion range, quick analysis time, cost-effectiveness for analysis and instrument maintenance, and the capability to analyse solid and liquid samples directly. While TXRF is not widely used in bioanalytical applications, it is increasingly gaining popularity. Research by Kubala-Kukus et al. [21] and Szoboszlai et al. [3] provides a comprehensive overview of the bioanalytical applications of TXRF. The reference work by Klockenkämper and Von Bohlen [19] focuses on estimating the critical thickness and sensitivities to work in the TXRF region for different matrices. In biosamples, the theoretical limit for thickness is 12 μm, and the theoretical maximum cover dry mass is 250 μg/cm^2^ for adequate analytical quantification and intensity-concentration linearity. For biomedical sample analysis, Lué M. Marcó and Hernández-Caraballo [22] investigated several sample preparation and calibration methods to facilitate direct analysis. These included slurry sampling, Compton peak standardisation, in situ microwave digestion, in situ chemical modification, and direct analysis with internal standardisation, applied to amniotic fluid, serum, urine, and brain biomaterials.

### 2.1. Microtomic Sections

One of the earliest applications of TXRF in the biomedical field was its use for the direct analysis of microtomic sections of biological and plant tissues, as developed by Von Bohlen et al. [23] and Klockenkämper et al. [24]. The samples were prepared using a freezing microtome to obtain sections approximately 10 microns thick with a wet mass of around 200 micrograms. These were directly deposited onto a quartz reflector and spiked with 10 nanograms of Ga as an internal standard. The method was applied to various foodstuffs, including nuts, mushrooms, and shrimp, as well as tissues such as liver and human lung. Quantitative results were obtained for elements such as Zn, Mn, Fe, Cu, Br, As, Sr, Rb, Se, and Ni, with concentrations ranging from 200 ppb to 700 ppm. More recently, Magalhães et al. [25] extended this methodology to study the elemental distribution of P, S, Cl, K, Ca, Cr, Mn, Fe, Ni, Cu, Zn, Se, Br, Rb, Sr, and Pb in both normal and cancerous tissues, particularly in the colon, breast, and stomach.

### 2.2. Human Fluids

#### 2.2.1. Blood Serum

Total Reflection X-ray Fluorescence (TXRF) has proven to be an exceptionally valuable analytical tool for blood analysis, particularly in detecting trace elements and heavy metals at ultra-low concentrations. Ayala et al. [26] conducted one of the first studies on analysing lead (Pb) in blood using TXRF, focusing on two groups of donors: one occupationally exposed to lead in a car battery factory and another unexposed group. The analysis involved adding 5 ppm of Sr as an internal standard, depositing 2 μL of the sample on a quartz reflector, and ashing it in a low-temperature oxygen plasma. Detected elements in the blood included K, Ca, Ti, Cr, Fe, Ni, Cu, Zn, Pb, Rb, and Sr, with a detection limit of 30 ppb for Pb. Donors exposed to occupational lead contamination showed significantly higher lead concentrations (230–680 ppb) compared to unexposed individuals, whose concentrations were below 100 ppb. Savage et al. [27] further explored the use of chemical modifiers in TXRF analysis of blood plasma, detecting elements such as As, Br, Cd, Ca, Cl, Co, Cu, I, Fe, Pb, Mn, Mo, Ni, Se, Sn, and Zn at concentrations ranging from 1 ppb to 270 ppm.

Determining nutrition-relevant elements such as Fe, Cu, Zn, and Se is a common analytical task in clinical laboratories. Stonach and Mages [28] demonstrated that TXRF could be effectively used for routine analysis of these dietary elements in human blood and serum by simply diluting the samples and applying internal standardisation. Majewska et al. [29] conducted a recent study to investigate element concentrations in human serum, focusing on P, S, Cl, K, Ca, Cr, Fe, Cu, Zn, Se, Br, Rb, and Pb, and establishing reference values for these elements. Similarly, Marguí et al. [30] developed an optimised, fast, sustainable, and reliable TXRF method for multielement analysis of whole blood samples. This optimised method was applied to blood samples from healthy volunteers (controls) and patients with thyroid gland disorders. Preliminary findings indicated significantly higher Zn and Br levels in some patients, though further studies are required to explore the relationship between these elements and thyroid diseases. Pierzak et al. [31] used TXRF to analyse chromium, selenium, and bromine concentrations in blood serum samples from 50 patients receiving parenteral nutrition. For comparison, serum samples from 50 patients without nutritional disorders served as a control group. The study concluded that TXRF is a reliable technique for monitoring trace element concentrations in human serum, particularly for patients undergoing parenteral nutrition therapy.

In the biomedical field, TXRF has been applied to predict the survival rate of COVID-19 patients by analysing the biomarkers Zn and Se in human serum [32]. Combining serum concentrations of Zn and selenoprotein P with patient age as a composite parameter for trace element metabolism offers valuable prognostic insights. Reference-range concentrations are associated with a high likelihood of survival, while significant deficiencies raise concerns and suggest the need for supplementation. However, it remains unclear whether correcting severe trace element deficiencies in critically ill patients aids in COVID-19 recovery, underscoring the need for further research.

More recently, Manjunatha et al. [33] have evaluated blood samples from diabetic adults. A total of 208 whole blood samples from diabetic (*n* = 104) and non-diabetic (*n* = 104) adults across various age groups were analysed using TXRF spectrometry with a sample dilution method. Statistical analysis was performed to assess descriptive statistics and determine a significant correlation between elemental concentrations in the blood samples of diabetic and non-diabetic adults. The mean concentration of nutritional-related trace elements in diabetic blood was as follows: Fe (46 +/− 5) > Zn (1.28 +/− 0.14) > Cu (0.10 +/− 0.01) > Cr (0.05 +/− 0.004) > Se (0.013 +/− 0.001) in mg/L, respectively. This study investigated the influence of nutrition-related trace element concentrations across various age groups, such as 25–40 years (young adults), 41–55 years (middle-aged adults), and 56–70 years (older adults). In this investigation, Zn and Cr concentrations differed significantly between diabetic and non-diabetic adults aged 56–70. These findings could help us to understand age-dependent changes in element concentrations, clarify their role in diabetes, and improve risk factor management associated with diabetes.

#### 2.2.2. Amniotic Fluid

Carvalho et al. [34] used TXRF to study human amniotic fluid and placenta to correlate metal content with newborn weight and maternal age. Very low levels of Ni and Sr were found in the amniotic fluid samples, and their concentrations were independent of both the mother’s age and the child’s weight. While Zn levels did not show significant differences across the samples, they were weakly related to birth weight. In contrast, Ca and Fe levels significantly correlated with maternal age and newborn weight. Marguí et al. [35] applied TXRF as a cost-effective, multielemental analytical method for analysing human placenta and amniotic fluid. The study compared a simple and rapid sample preparation method (suspension) with acid digestion and ICP-AES procedures. Detection limits for most elements were in the low mg/kg range for both sample treatments. Accurate and precise TXRF results were achieved using internal standardisation for quantification, along with a correction factor to compensate for absorption effects.

Recently, Hauser et al. [36] applied TXRF to the placenta quantitation of relevant elements because the placental elemental composition can indicate neonatal health. Medical studies that reveal cause-and-effect relationships or monitor potential environmental influences consist of large data series to ensure statistically sufficient data. TXRF spectrometry allows the analysis of minute sample amounts to be directly conducted. They report a method to prepare sample suspensions for an adequate and fast TXRF analysis of large sample series. Applying this method, possible effects on the fixation time and the sampling location, i.e., the maternal versus foetal side of the placenta, were studied. Significant differences in foetal placenta tissue compared to maternal or intermediate tissue were observed, revealing an accumulation of some trace elements in the foetal side of the placenta.

#### 2.2.3. Cerebrospinal Fluid

It is well-known that trace elements play an essential role in the human central nervous system. Significant variations in the concentration of trace elements in body fluids may occur in neurodegenerative diseases. Ostachowicz et al. [37,38] studied the cerebrospinal fluid by TXRF, particularly in the neurodegenerative disease amyotrophic lateral sclerosis (ALS). Samples of the cerebrospinal fluid from two groups of patients, ALS and control, were analysed. Cl, K, Ca, Cr, Mn, Fe, Ni, Cu, Zn, Rb, and Br were evaluated. The study showed differences in the concentrations of Zn and Cl between the ALS and the control group.

#### 2.2.4. Oral Fluid

TXRF also evaluated oral fluids, and one of the first studies was made by Abraham et al. [39]. This work presents the study of the elemental composition of oral fluids, such as saliva and gingival crevice fluid, and their relation with smoking. Two sets of patients, smokers and non-smokers, were selected according to specific criteria to analyse saliva and gingival crevice fluid. The most significant differences in concentration between smokers and non-smokers were found in saliva samples for S, K, and Ca. More recently, the same group of Abraham et al. [40] applied TXRF spectrometry in an indirect study of corrosion of dental implants by analysing changes in metals’ elemental concentration in oral fluids. Degradation of the implant surface releases material to the medium, which, depending on the concentrations, can represent a toxic risk, organic malfunction, pain, rejection, and more. The concentrations of representative metals such as Ti, Al, and V in saliva and gingival fluids were analysed, employing total reflection X-ray fluorescence analysis using synchrotron radiation to evaluate the degradation process.

Recently, Zambianchi and Zambianchi have quantified the salivary iodine levels as biomarkers [41]. Iodine is a vital trace element crucial for synthesising thyroid hormones and regulating metabolism and tissue growth. Recent studies have shown a strong correlation between urinary iodine content and iodine present in saliva. So, using Monte Carlo simulations, the authors present a TXRF-based method to quantify salivary iodine biomarkers for assessing and monitoring iodine status. Of clinical significance, quantifying salivary iodine using TXRF to assess and monitor iodine status could be particularly helpful to patients undertaking ^131^I treatment. In addition, it could be used in epidemiological field studies to determine iodine deficiency/excess in children and pregnant women.

#### 2.2.5. Urine

One of the first applications of TXRF to the study of urine fluid was developed by Zarkadas et al. [42]. In this reference work, TXRF determined trace uranium content. The method utilises open vessel digestion with nitric acid and uranium preconcentration by using sodium dibencyldithiocarbamate (NaDBDTC) as the complexing agent. Detection limits around one ppb were achieved, with recoveries close to 100% and uncertainties under 10%. Telgmann et al. [43] developed a simple and rapid TXRF method for determining gadolinium (Gd) concentrations in urine and blood plasma samples. The limits of detection (LOD) were 100 μg/L for urine and 80 μg/L for blood plasma, while the limits of quantification (LOQ) were 330 μg/L for urine and 270 μg/L for blood plasma. This TXRF methodology allows for the analysis of urine samples from MRI patients up to 20 h after the administration of Gd-based contrast agents. Thus, combining urine analysis with blood Gd evaluation enables the monitoring of Gd excretion kinetics from the patient’s body. Majewska et al. [44] applied TXRF to establish reference values for elemental concentrations in human urine. Certified human urine samples and real samples were analysed, with all sample preparation parameters and measurement conditions carefully optimised. The optimal preparation method and conditions were defined, leading to the determination of reference values for several elements (K, Ca, Cr, Mn, Fe, Ni, Cu, Zn, Br, Rb, and Sr) based on the analysis of 100 urine samples. Key findings included higher concentrations of Ca, Cr, Zn, Rb, and Sr in men’s urine compared to women’s urine; lower Mn concentrations in men’s urine than in women’s; and lower levels of Zn, Rb, and Sr in both older men and women compared to younger individuals.

Recently, Jasna Jablan et al. [45] evaluated human exposure to toxic metals and metalloids due to the significant public health concern and because they can seriously affect human health. The study aimed to establish the presence of toxic metals and metalloids in the urine of 21 non-professional athletes who participated in a mountain ultramarathon. Urine samples were collected at four time points: at the beginning of the race (pre-race samples), immediately after, and 12 and 24 h post-race. The collected urine samples detected Al, As, Ba, Ni, Pb, Rb, Sr, V, and Tl. Changes in the urine composition were observed over time, with increasing average urinary metals and metalloid levels. Nevertheless, only two significant results were observed: an increase in As and Rb. The results indicate a high degree of inter-subject variability. The results obtained show that the content of toxic metals and metalloids increases in the urine samples collected after the race, which could confirm the statement that physical activity can increase the excretion of toxic metals and metalloids from the body.

#### 2.2.6. Seminal Fluid

Camejo et al. [46] were the first to apply TXRF in the study of human sperm, specifically to determine the concentrations of Se, Cu, and Zn in the semen of patients with varicocele and explore their relationship with seminal parameters. The study found that a decrease in selenium concentration was linked to a decline in seminal parameters, including sperm concentration, motility, and morphology. Marguí et al. [47] developed a simple and reliable method for determining several elements in seminal plasma samples using TXRF. Under optimal analytical conditions, the detection limits for trace elements ranged from 0.04 to 0.3 mg/kg. The accuracy and precision of the results, evaluated through spiked seminal sample analysis, were generally acceptable, with recovery values ranging from 87% to 109% and relative standard deviations between 3% and 12% (*n* = 5). Among the trace elements studied for their role in the antioxidant defence system, only Zn was quantifiable, and some differences in Zn concentrations were observed across the studied groups.

More recently, Margui et al. [48] have critically evaluated the possibilities and limitations of simple sample preparation (i.e., dilution and suspension) combined with TXRF spectrometry to analyse different biological samples, including seminal plasma. The main conclusion is that a simple sample preparation by diluting with water or with a diluted solution of a surfactant, in combination with TXRF analysis, generates acceptable and valuable results that open the applicability of TXRF spectrometry in the clinical determination of minor and trace elements relevant in the field of medical diagnostics.

### 2.3. Metalloproteins

Wittershagen et al. [49] developed a method using TXRF to determine metal cofactors in respiratory chain complexes. The two-terminal oxidases—cytochrome c oxidase and quinol oxidase—were isolated from the soil bacterium Paracoccus denitrificans and transferred from their standard saline buffer to a solution containing 100 mmol/L tris(hydroxymethyl)aminomethane acetate (TRIS) with 0.02% Triton X. This approach enhanced the signal-to-noise ratio, enabling accurate evaluation of elements such as Fe, Ni, Cu, Zn, Mn, and Mo without decomposition. Additionally, sulphur content in protein samples could also be reliably determined. Wellenreuther et al. [50] investigated the best conditions for the analysis by TXRF of the following metalloproteins: hELAC1, insulin, concanavalin A, thermolysin, and glucose isomerase. The study concluded that elaborate sample decomposition is not generally necessary; direct analysis of protein samples is feasible. More recently, Strohmidel et al. [51] investigated the binding of ethylmercury (EtHg+) released from the preservative thiomersal by hydrolysis to proteins in influenza vaccines via ultrafiltration and subsequent TXRF analysis as well as size exclusion chromatography (SEC) hyphenated to inductively coupled plasma-mass spectrometry (ICP-MS). Hegde et al. [52] evaluate autism as a heterogeneous neurodevelopmental disorder. Human homeostatic iron regulator (HFE) codes for HFE protein were evaluated because HFE protein is essential for inhibitory regulation of the endocytosis of iron. The present contribution aims to screen C282Y and H63D polymorphisms of the HFE gene in autistic children. So, 30 autistic children and 30 healthy age-matched control children were evaluated. TXRF analysis was performed to quantify Fe in plasma. Genomic DNA was extracted using peripheral blood samples, and targeted SNPs were screened using restriction fragment length polymorphism. Genotype, allelic frequencies, and risk ratio were calculated using the statistical method. TXRF analysis shows a significantly low concentration of iron in autistic children compared to the control group.

Recently, Hadrian et al. [53] have studied the effect of primary copper metabolism disturbance on the organs’ elemental, protein, and lipid composition in Jackson’s toxic milk mouse. Toxic milk (txJ) is an autosomal recessive mutation in the Atp7b gene in the C3H/HeJ strain, observed at the Jackson Laboratory in Maine, USA. TxJ mice exhibit symptoms similar to those of human Wilson’s disease (WD). The study aimed to verify organ involvement in a mouse model of WD. TxJ mice and control animals were sacrificed at 2, 4, 8, and 14 months of age. TXRF, next to FTIR and micro-XRF, was used to determine the elemental concentration in organs. Elevated concentrations of Cu were observed in the liver, striatum, eye, heart, and duodenum of txJ mice across age groups. The distribution of Cu deposits in brains was uniform, with no prevalence in any anatomic structure in either group, but significant protein structure changes were observed exclusively in the striatum of txJ. In this txJ animal model of WD, pathologic copper accumulation occurs in the duodenum, heart, and eye tissues. Increased copper concentration in the liver and brain increases saturated fat content and disturbances in secondary protein structure, leading to hepatic injury and neurodegeneration.

### 2.4. Cytosol Pellet

Günther et al. [54] introduced a novel methodology for the first TXRF approximation to the intracellular speciation in plant cells. Using TXRF, they simultaneously quantified Ca, Cu, Fe, K, Mn, Rb, Sr, and Zn in 12 different vegetable foodstuffs and their cellular fractions after mechanical cell disruption. The homogenates were separated into cytosol (liquid fraction) and pellets (solid fraction) via centrifugation. Before TXRF analysis, the samples were digested with nitric acid and spiked with Ga as an internal standard. The study revealed the elemental distribution within each fraction, showing that, on average, Sr, Ca, and Fe were predominantly associated with the pellet components.

In a related study, Gonzalez et al. [55] utilised TXRF for the first time to determine Cu, Fe, Zn, Ca, and S in various mammalian tumour cell lines. These included human clonal cell lines (HepG2, Caco-2, HeLa), mouse clonal cell lines (NIH 3T3, N2A), and a rat clonal cell line (B12). The research evaluates the elemental distribution, the intracellular concentrations, and the changes in their proportions after chronic copper exposure. Results demonstrated that TXRF could detect total trace metal content with a minimal number of cells (1–2 × 10^6^), while 4–6 × 10^6^ cells sufficed to determine the cytosol/pellet distribution.

Lossow et al. [56] recently evaluated the trace elemental profile in murine liver tissue samples, comparing ICP-MS/MS and TXRF techniques. Today, the trace analysis of small sample amounts may be a big challenge, especially if also endpoints want to be addressed in the same sample. This work examined trace elements (iron, copper, zinc, and selenium) in murine liver tissue prepared by a RIPA buffer-based lysis method. So, after centrifugation, lysates and pellets were obtained, and trace elements were analysed with TXRF in liver lysates. The results were compared to those obtained by a standard microwave-assisted acidic digestion with subsequent ICP-MS/MS analysis of the same liver tissue, liver lysates, and remaining pellets. In addition, trace element concentrations, determined in murine serum with both methods, were compared. For serum samples, both TXRF and ICP-MS/MS provide similar and highly correlating results. Furthermore, in liver lysate samples prepared with RIPA buffer, comparable trace element concentrations were measured by TXRF as with the standard digestion technique and ICP-MS/MS. Only marginal amounts of trace elements were detected in the pellets. The results obtained by the present study indicate that the RIPA buffer-based method is suitable for sample preparation for trace element analyses via TXRF, at least for the murine liver samples investigated here.

### 2.5. Cancer Research

#### 2.5.1. Antitumoural Compounds Action

Fernández-Ruiz et al. [57] applied TXRF to monitor the molecular introduction kinetics of the Pt-Berenil and cis-DDP compounds when crossing the biological barrier of the HeLa cells. Medicines that interact with nuclear DNA were also quantified in this study. This work marked the beginning of intracellular metal studies combined with effective subcellular constituent separation using TXRF. Using a sample volume of just 100 μL, Pt concentrations ranging from 3 to 30 ng/mL were measured with a relative standard deviation of 2% to 8%. Applying previous methodology, Perez et al. [58] used TXRF spectrometry as a tool to study the cellular uptake, DNA binding, and apoptosis induction of cytotoxic trans-(PtCl2(N,N-dimethylamine)(isopropylamine)) in A2780cisR ovarian tumour cells. The results show that this novel drug was more cytotoxic and induced more apoptotic cells than cisplatin in A2780cisR cells. However, the intracellular accumulation and extent of binding to the DNA of trans-(PtCl2(N,N-dimethylamine)(isopropylamine)) were lower than that of cis-DDP.

More recently, Majer et al. [59] conducted a similar study on dinuclear Rh(II) complexes of phenylalanine derivatives as potential anticancer agents for colon carcinoma. Using TXRF spectrometry, they quantified the intracellular rhodium content in human colon HT-29 cells, which ranged from 25 to 2500 ng per 10^6^ cells, depending on the type of ligand and its coordination number.

#### 2.5.2. Tumoural/Cancerous Tissues

The use of TXRF for studying healthy and cancerous tissues has been widely explored. One of the earliest studies in this field was conducted by Czarnowski et al. [60] in 1997, aiming to develop a novel methodology for cancer diagnosis. This pioneering research analysed trace element distributions in carcinomas of the digestive tract compared to normal tissues from the human stomach, colon, and rectum, correlating findings with cancer types. Significant reductions in Cr, Fe, and Ni were observed in stomach carcinomas; Cr and Co in colon carcinomas; and notable accumulations of K in colon cancer tissues and Fe and K in rectal neoplastic tissues, albeit in a small patient cohort. Li et al. [61] examined trace element variations in lung and cervical cancer cells before and after apoptosis using the TXRF line at the Beijing synchrotron. Similarly, Magalhães et al. [62] investigated the distribution of elements such as P, S, Cl, K, Ca, Cr, Mn, Fe, Ni, Cu, Zn, Se, Br, Rb, Sr, I, and Pb in normal and cancerous tissues from the same individuals. Thin tissue sections from carcinomas of the colon, breast, and uterus in seven German patients and rectum, sigmoid, thyroid, kidney, larynx, and lung tissues from ten Portuguese patients were directly analysed by TXRF. A consistent pattern emerged: carcinoma tissues exhibited increased or stable levels of P, S, K, Ca, Fe, and Cu, while Zn and Br levels were decreased compared to healthy tissues. Carvalho et al. [63] provided a comprehensive review of studies comparing elemental profiles of healthy and cancerous tissues—including breast, lung, serum, intestinal, prostate, and the uterus—using TXRF. Szoboszlai et al. [64] developed a direct method for elemental analysis of cancer cell lines by depositing tumourous or healthy cells onto TXRF reflectors. This approach was applied to human colon adenocarcinomas to measure Cu, Zn, and Fe levels. Leitão et al. [65] utilised TXRF to analyse the elemental profiles of P, S, K, Ca, Fe, Cu, Zn, and Rb in healthy and cancerous prostate tissues to explore diagnostic strategies. Lankosz et al. [66] applied TXRF to the elemental analysis of brain tumours of different types and grades of malignancy. The following elements were present in all the neoplastic tissues analysed: K, Ca, Fe, Cu, Zn, and Rb. The analysis results showed that the elemental composition of a relatively small tissue fragment represents satisfactorily the biochemical “signature” of a cancer. It was possible to differentiate between some types of brain tumours based on the element concentrations determined.

Recently, Olbrich et al. [67] and Wilk et al. [68] evaluated the TXRF technique’s analytical capability by developing a round-robin exercise between four European laboratories. The final idea was to apply the traceability of the TXRF to evaluate the glioblastoma (GBM) multiforme influence on the elemental homeostasis of the distant organs. GBM is a fast-growing and aggressive brain tumour that invades nearby brain tissue but generally does not spread to distant organs. Nonetheless, if untreated, GBM can result in patient death in time, even less than a few months from the diagnosis. This work evaluates the elemental abnormalities in selected body organs (kidney, heart, spleen, lung) in two rat models of GBM. The animals used for the study were subjected to the implantation of human GBM cell lines (U87MG and T98G) characterised by different levels of invasiveness. The comparison of the TXRF data obtained for animals subjected to T98G and U87MG cell implantation showed several elemental anomalies in the examined organs. Moreover, the abnormalities were found in rats even if neoplastic tumours did not develop in their brains. Most alterations for both experimental groups were noted in the spleen and lungs, with the opposite direction of the found element changes in these organs. The observed disorders of element homeostasis may result from many processes occurring in the animal body due to the implantation of cancer cells or the development of GBM, including inflammation, anaemia of chronic disease, or changes in iron metabolism. Tumour-induced changes in organ elemental composition detected in the four cooperating TXRF European laboratories collaborating in this study were usually in good agreement. Regarding elements with higher atomic numbers (Fe, Cu, Zn, and Se), 88% of the results were classified as fully compliant.

More recently, Hiremath et al. [69] investigated the photon interaction parameters to reveal cancer development in breast tissues at various ages. The present work estimated the mass attenuation coefficient (MAC) values using EpiXs software version 2.0.1 for normal and malignant human breast tissues at different age groups by incorporating experimentally quantified trace elements by TXRF spectrometry. A significant difference in the effective atomic number values was observed between normal and malignant breast tissues of different age groups, which is helpful in radiation diagnostics and therapeutics.

### 2.6. Vegetal Cell System

Mages et al. [70] utilised TXRF to analyse biofilms as bioindicators of water pollution. They observed concentration differences of several orders of magnitude between biofilms from polluted and unpolluted water sources. The study evaluated elements including K, Ca, Cr, Mn, Fe, Ni, Cu, Zn, As, and Sr, using sample masses between 10 and 500 μg and achieving detection limits in the ppb range. Woelfl et al. [71] applied TXRF to examine trace metals in planktonic microcrustaceans, analysing samples with masses under 50 μg. They quantified Mn, Fe, Ni, Cu, Zn, and As at ppm levels. Pepponi et al. [72] employed TXRF to study pollen as a bioindicator of atmospheric pollution, focusing on *Corylus avellana* L. (hazel) pollen collected from five sites in the province of Trento, Italy, with varying levels of human impact. Quantified elements included Al, P, S, K, Ca, Ti, V, Cr, Mn, Fe, Ni, Cu, Zn, Br, Rb, Sr, Ba, and Pb, with detection limits in the low ppb range.

Mosses have long been considered promising candidates for environmental pollution monitoring. One of the earliest TXRF studies in this area was conducted by Market et al. [73], who analysed moss samples from three species for trace elements. Approximately 20 elements were quantified, with detection limits typically ranging from 0.2 to 0.5 μg/g. The results revealed significant variations in pollution levels with hazardous metals, depending on the sampling site. Some samples demonstrated the feasibility of using TXRF for routine monitoring of plant samples. More recently, simplified methodologies such as suspension and internal standardisation of moss analyses, or the more traditional acid digestion, have been successfully applied in environmental pollution assessments. These include studies in the Toluca Valley of Mexico by Zarazua-Ortega et al. [74] and in Paris by Natali et al. [75]. Espinoza-Quiñones et al. [76] used TXRF to study the bioaccumulation kinetics of lead in the aquatic macrophyte *Salvinia auriculata*. The data indicated that both adsorption and bioaccumulation mechanisms were involved, with competition between phosphorus macronutrients and lead for plant growth when high lead concentrations were present in the roots. Höhner et al. [77] developed a rapid TXRF protocol for the simultaneous quantification of elements such as K, Ca, S, Mn, and Sr in *Arabidopsis thaliana* leaf specimens. TXRF is a powerful tool for investigating plant ion homeostasis, which is essential for vital biochemical processes like photosynthesis. This method provides detailed quantitative insights into (I) the effect of environmental stress on plant ion balance, (II) ion gradients between plant tissues, and (III) ion levels in plant mutants with impaired growth or heterogeneous phenotypes.

De Almeida et al. [78] evaluated the application of ED-XRF and TXRF spectrometries for vegetal mass-limited sample analysis, particularly for soybean roots and shoots. The proposed method’s trueness was verified by analysing several plant tissue-certified reference materials. Additionally, soybean samples were analysed using a validated total reflection X-ray fluorescence (TXRF) method, and good agreement was found between both analytical approaches. Singh et al. [79] have recently presented an interesting review related to the microchemical imaging applications by XRF, μ-XRF, μ-SRXRF, and TXRF, covering a wide variety of plant tissues such as leaves, roots, stems, and seeds. This work shows the analytical power and limitations that these types of spectrometries present for vegetal tissue elemental analysis.

### 2.7. Microbiological Systems

Janik et al. studied by TXRF [80] the composition of spores, peridium walls, and lime nodes of *Physarum compressum sporocarps*, cultivated on rabbit dung as a natural growing environment for the slime mould and on artificial agar medium with the primary goal of evaluating differences that may be dependent on substrates. According to TXRF results, the fruiting bodies from the agar medium revealed lower concentrations of K, Ca, Cr, Mn, and Fe concerning fruiting bodies from the dung, reflecting elemental relationships in the experimental media. Fernández-Ruiz et al. [81] utilised TXRF spectrometry to study the kinetics of Cr(VI) bioaccumulation in the bacterium ANCR (*Acinetobacter beijerinckii* type). The results demonstrated that this novel bacterial strain effectively reduces chromium in the culture medium, highlighting its potential as a promising Cr(VI) bioremediation agent in polluted wastewater. Furthermore, the study showcased the versatility, sensitivity, and analytical power of TXRF spectrometry for evaluating metals in microbiological systems. Fiedor et al. [82] applied TXRF spectrometry to comprehensively analyse trace element content in purple non-sulphur phototrophic bacteria, focusing on their chromatophores and selected photosynthetic structures under varying oxygen growth conditions. The study addressed the lack of consistent data on microelement content, distribution, and inter-element correlations. Using *Rhodobacter sphaeroides* as the model organism, qualitative analysis identified microelements not typically considered essential to the bacterial ionome. Quantitative results emphasised Fe as the central trace element in this phototrophic species, regardless of growth conditions or sample type. An ionomic approach, supported by statistical analysis, uncovered intriguing inter-element relationships within cells and phototrophic membranes. This research underscores the vast potential and utility of TXRF for a wide range of biological and environmental applications.

Recently, Augustynowicz et al. [83] used the TXRF to evaluate the benefits and limitations of the application of *Callitriche cophocarpa* Sendtn. (water-starwort) to remove Cr under real-world conditions. Cr content in plant organs at the contaminated sites was 33 up to 83 times greater than in the control leaf/stem and roots, respectively. As the main conclusion, the evaluated plant water-starwort can be considered a potential candidate for Cr bioremediation under controlled conditions.

### 2.8. Pharmacological Compounds and Drugs

Wagner et al. made one of the first works where TXRF was applied to pharmacological compounds [84]. In this work, they applied TXRF to determine trace metals in drugs. Various samples of lecithin, insulin, procaine, and tryptophan from different origins were investigated. The elemental profile and concentrations provide samples of fingerprints, which offer the possibility of differentiate and discriminating between different batches of the analysed substances originating from different production or purification processes. TXRF facilitates the characterisation of such samples without extensive pre-treatment and provides fast multielement determination of elements with atomic numbers from Z = 14 to Z = 92 based on matrix-independent quantification. Muratsu et al. [85] applied TXRF to the trace elemental analysis of illicit methamphetamines. So, 50 kinds of seized methamphetamines were analysed, half of which had been classified as pure and the remainder as impure. As trace elements, Br, Hg, I, Ca, Fe, Cr, Mn, Ni, Cu, and Zn were detected. Among them, Br was detected in all samples, and its content was distributed between 0.4 ng and 71 ng per 1 mg of methamphetamine KCI. Hg and I were probably to be derived from synthetic reagents. Iron and other heavy metals could be derived from impurities in synthetic solvents or metal containers through synthetic or smuggling processes. Elements detected in seized methamphetamines are applicable as tracing markers for the ascertainment of clandestine synthetic techniques or places to make illicit methamphetamine salts. Greaves et al. [86] apply TXRF for quality control of chemotherapy drugs. So, they developed a method employing Compton peak standardisation and matrix-matched spiked samples with TXRF to determine platinum plasma concentrations of patients undergoing chemotherapy with Pt-bearing drugs. Direct blood plasma analysis attains Pt detection limits of 70 ng/mL. The work demonstrates the potential abilities that TXRF possesses for pharmacokinetic studies or the routine optimisation and quality control of Pt chemotherapy treatments. Borgese et al. [87] used TXRF for the investigation of heavy metal poisoning due to the non-adequate use of a traditional Ayurvedic drug. Subsequently, an Indian person treated with a traditional preparation of mineral powder for urological problems showed clinical signs of heavy metal poisoning. TXRF performed a metal profile analysis to demonstrate and confirm the causal relationship between the disease and the use of the drug. TXRF analysis was made by over-reflector direct digestion with nitric acid of 1 cm of patient hair. Results confirmed that the presence of a high concentration of Pb in the patient’s hair correlated with the presence of this toxic metal in the Ayurvedic drug preparation. Antosz et al. [88] introduced TXRF to the pharmaceutical industry, demonstrating its effectiveness for trace element analysis. The study showed that TXRF provided results comparable to those obtained by ICP-MS for measuring Pd and Cu in the same samples. Statistical analysis confirmed that the results from both techniques were equivalent at a 95% confidence level.

Recently, Danilov et al. [89] developed several sample preparation methods for determining the elemental composition of vitamin–mineral complexes using TXRF spectrometry. TXRF results were compared with ICP-AES, and although TXRF showed lower agreement factors than widely used ICP-AES, it uses a much simpler sample preparation technique (dissolution and suspension). As a consequence, it shortens the analysis time by several times.

### 2.9. Nanoparticles in Biomedicine

Magnetic nanoparticle suspensions in aqueous media have garnered significant interest due to their potential applications in biomedicine, including biomolecule separation, magnetic resonance imaging (MRI), and drug delivery [90]. Fernández-Ruiz et al. [91] developed a TXRF-based procedure to analyse Fe and trace metals in functionalised magnetite nanoparticles, used as contrast agents for MRI, without requiring sample digestion. This direct solid-state approach streamlined the analysis process. Gold nanoparticles (AuNPs) also hold great promise in biomedical applications, though their impact on the human body remains insufficiently understood. Fernández-Ruiz et al. [92] conducted one of the first studies to evaluate gold nanoparticles (AuNPs) using TXRF, focusing on the bioaccumulation kinetics of gold nanorods (AuNRs) after intravenous administration in mice. They analysed the accumulation of AuNRs in major organs such as the liver, spleen, brain, and lungs at various time points and assessed their elimination kinetics via urine samples. The study identified two distinct behaviours: AuNRs rapidly accumulated in highly vascular filtration organs, such as the liver and spleen, but showed no significant bioaccumulation in the brain and lungs during the observation period. This work highlighted TXRF’s power, versatility, and precision in evaluating AuNPs in biological systems and its broader applicability to metallic nanoparticles. Mankovskii and Pejović-Milić [93] further advanced TXRF’s capabilities by validating a method for quantifying trace-level AuNPs in organic matrices. Their study investigated appropriate internal standards, fitting methodologies, and sample preparation techniques, ensuring robust and accurate nanoparticle analysis. The developed method was validated with reference material nanoparticles. Recovery rates of (102.7 ± 3.7)% and (100.9 ± 5.1)% were achieved for nanoparticles in an ionic solution and organic matrix, respectively. These results suggest that TXRF is a good technique to accurately quantify gold nanoparticle uptake in cancer cells. In this line, Mankovskii and Pejovic-Milic [94] applied TXRF to quantify gold nanoparticles in histologically thin tissue slices. The promise of gold nanoparticles (AuNPs) in cancer applications remains an active area of research. The experimental TXRF results revealed nearly 100% quantification recovery of AuNPs in all permutations of sample configuration, making TXRF a viable option for assessment of tumoural AuNP uptake with minimal sample preparation. Zambianchi et al. [95] applied TXRF to the quantification of gold using the Monte Carlo simulation (MCNP code) applied to cancer cell research. As the main conclusion, for stable colloidal samples of AuNPs, the signal depends on the Au mass only, being statistically the same as the acid-digested cases. The results extend TXRF spectroscopy as a tool to quantify the uptake of AuNPs by cancer cells. So, a potential novel application of TXRF to circulating tumour cell (CTC) detection is proposed. Pernas-Pleite et al. [96] applied TXRF, among other analytical techniques, to control the green extracellular synthesis of silver nanoparticles (AgNPs) by Pseudomonas alloputida. Bacterial resistance to antibiotics is on the rise and hinders the fight against bacterial infections, which are expected to cause millions of deaths by 2050. New antibiotics are difficult to find, so alternatives are needed. One of these alternatives could be metal-based drugs, such as silver nanoparticles (AgNPs).

Recently, Aziz et al. [97] used TXRF to control the synthesis and evaluation of the antibacterial and antifungal activities of CuO-ZnO-Co_3_O_4_ nanocomposites. In this study, the antibacterial activity of CuO-ZnO-Co_3_O_4_ nanocomposites against Gram-negative bacteria (*Escherichia coli*, *Klebsiella pneumoniaea*, pseudomonas, and Salmonella) and Gram-positive bacteria (*Staphylococcus aureus*) was investigated and compared with the antibiotic azithromycin. In addition, the effect of the nanocomposite on fungi was studied and compared with the antifungal myostatin.

### 2.10. Metals in Molecular Layers

Zheludeva et al. [98,99] were the first to utilise the angle dependence of the X-ray standing wave (XSW) field to study molecular monolayers. This approach allowed the localisation of ions within the monolayer relative to the film-liquid interface by analysing the angular curves of their characteristic fluorescence radiation around the critical angle. The method was applied to phthalocyanine, cyclolinear polyorganoxilanes, phospholipids in Langmuir layers on liquid surfaces, and a protein–lipid film based on Ca-ATPase on a solid substrate. This pioneering work opened up a new field for angle-dependent TXRF in studying the diffusion of metals at system interfaces. More recently, Brücher et al. [100] employed angle-dependent TXRF in conjunction with XSW and streaming current measurements to evaluate functionalised solid-liquid interfaces. Their study focused on surface charges, interfacial potential, and ion distributions. Thin films of an aqueous solution containing Br^−^ anions and Fe^3+^ cations at a concentration of 10 mg/L were prepared on functionalised silicon wafers. The ion distribution was measured with nanometre resolution, distinguishing between absorbed and mobile ions on the surface and in the diffusive layer.

## 3. Conclusions

TXRF has proven to be an extraordinarily versatile and promising analytical tool in the biological, biomedical, biochemical, pharmacological, and molecular fields. Its microanalytical ability to detect elements at trace levels, combined with its low instrumental and maintenance cost, makes it an accessible and powerful option for addressing complex issues in these scientific fields. Specifically, TXRF holds great potential in bioanalytical research as it allows for analysing essential elements in biological systems, facilitating the study of processes at the cellular and molecular levels.

In the biological and biomedical fields, TXRF offers the ability to study metallomic interactions and their implications for human health, enabling detailed analysis of metal distribution and its impact on diseases related to metal imbalances. For example, in studying conditions such as Alzheimer’s disease, cardiovascular diseases, or cancer, the technique can help identify disruptions in trace metal levels that could indicate these disorders.

In the pharmacological field, TXRF can play a crucial role in evaluating drug release and distribution within the body. Its ability to study the concentration of metals and trace elements in biological samples such as blood, tissues, and bodily fluids allows for detailed studies on drug bioavailability and pharmacokinetics. Additionally, it can be used to monitor drug toxicity, helping to identify side effects associated with the accumulation of heavy metals or the uncontrolled release of certain compounds.

In the molecular field, TXRF facilitates the understanding of mechanisms at the cellular level, enabling the analysis of the elements involved in fundamental biochemical processes such as protein synthesis, cellular metabolism, or cellular stress responses. Analysing metals in cellular chemical equilibrium is essential for understanding how these elements interact with biomolecules and participate in vital processes. Furthermore, by applying it to complex biological systems and difficult matrices, TXRF provides an accessible and precise approach to investigating new therapies and treatments.

From an analytical point of view, TXRF provides exceptional sensitivity for trace element detection, often down to the nanogram or even the picogram range. These detection limits are comparable to techniques like ICP-MS, but TXRF requires smaller sample volumes and more straightforward preparation. TXRF is a microanalytical technique and requires only microlitre or microgram quantities of the sample, making it ideal for applications where sample availability is limited, such as in biological or clinical studies. Techniques like ICP-MS or AAS often require significantly larger volumes.

TXRF significantly reduces matrix effects compared to conventional XRF due to its total reflection geometry, enabling linear, more accurate, and reliable results for samples with complex matrices. TXRF instruments are generally less expensive to purchase, operate, and maintain compared to techniques like ICP-MS or AAS. This fact makes TXRF an accessible option for smaller labs and organisations. TXRF requires minimal chemical treatment of samples, and in many cases, samples can be analysed directly after being placed on a carrier substrate. This simplicity contrasts with the extensive sample digestion required for techniques like ICP-MS or AAS.

TXRF enables simultaneous detection of multiple elements in a single analysis, providing a broad elemental profile without requiring sequential measurements. Techniques like AAS are typically limited to single-element detection per analysis. Unlike ICP-based methods that require complex external calibration standards, TXRF often uses simple internal standardisation, streamlining the quantification process.

TXRF can analyse solid, liquid, and powdered samples with minimal adaptation, providing flexibility for diverse applications. Other methods often specialise in specific sample states. TXRF typically avoids the need for large quantities of reagents or consumables, reducing chemical waste compared to ICP-MS or AAS, which require acids and other reagents for sample digestion. With relatively quick sample preparation and analysis, TXRF allows for rapid results compared to techniques like ICP-MS, which may require time-intensive sample digestion and instrument setup.

TXRF excels in analysing samples with challenging matrices, such as biological fluids, tissue extracts, or pharmaceutical formulations, without extensive pre-treatment.

On the other hand, TXRF presents some limitations and drawbacks with respect to other, more conventional analytical techniques. TXRF presents a limited sensitivity for light elements. While TXRF is highly sensitive for trace-level detection, its performance is limited for elements with low fluorescence yields, such as light elements with Z < 12. Techniques like inductively coupled plasma mass spectrometry (ICP-MS) or atomic absorption spectroscopy (AAS) are better alternatives for such elements.

If throughput for large sample sets is considered, techniques like ICP-MS, which can be automated for large sample sets using autosamplers and automatic injectors, often provide better efficiency than TXRF. TXRF typically requires individual sample preparation, deposition, and analysis, making it less suitable for high-throughput scenarios. Nevertheless, the last commercial TXRF instruments are also equipped with automatic autosamplers with up to 90 samples (Bruker S4 TStar).

TXRF has a sample preparation constraint because it requires the deposition of the sample, including creating a thin, uniform layer on a carrier substrate to minimise matrix effects. This step can be challenging for complex or heterogeneous samples, whereas other techniques (e.g., ICP-MS or AAS) often have broader tolerance for sample conditions.

TXRF does not provide isotopic information, which is critical in studies involving isotopic labelling or radiometric analysis. ICP-MS or mass spectrometry (MS)-based methods are indicated for such purposes.

Table 1 shows a technical comparison of TXRF versus related techniques, resuming objectively its benefits, limitations, and drawbacks.

In conclusion, the use of TXRF in the biological, biomedical, pharmacological, and molecular fields continues to expand and demonstrate significant potential for solving complex problems related to cell biology, pharmacokinetics, and toxicology. Its ability to provide accurate and detailed information about elements present in biological samples makes it an invaluable tool in disease research, drug development, and understanding biochemical processes at the molecular level. As the technique evolves continuously, its applicability in these fields can only grow, opening new opportunities for advancing biomedical and molecular science.

## Figures and Tables

**Table 1:** Comparative table outlining the pros and cons of TXRF (total-reflection X-Ray fluorescence) against related techniques, such as EDXRF (energy dispersive X-ray fluorescence), WDXRF (wavelength dispersive X-ray fluorescence), and ICP-MS (inductively coupled plasma mass spectrometry).

Feature	TXRF	EDXRF	WDXRF	ICP-MS
Detection Limit	Low (sub-ppb level achievable for some elements)	Moderate (ppm range)	Low to Moderate (ppm range)	Ultra-low (ppt to sub-ppt range)
Quantitative Analysis	Excellent for small sample volumes, requires internal standard for precise quantification	Good, requires calibration curves	Very accurate, robust quantification with calibration curves	Highly accurate, requires complex standards and calibration
Elemental Range	Z > 11 (sodium to heavier elements)	Z > 11 (sodium to heavier elements)	Z > 4 (beryllium to heavier elements)	Nearly all elements, including isotopes
Sample Preparation	Minimal, often just drying a drop of liquid sample	Minimal, but matrix effects are more pronounced	Requires pressed pellets or fused beads	Extensive, including acid digestion or dilution
Sample Volume	Very small (microlitre scale)	Small to moderate	Moderate	Moderate to large
Matrix Effects	Minimal (due to total reflection geometry)	Significant	Moderate	Minimal (after digestion)
Instrumentation Cost	Moderate	Low to moderate	High	Very high
Operational Cost	Low (no consumables except standards)	Low	Moderate	High (argon gas, acids, standards, etc.)
Ease of Use	Simple, user-friendly, often portable	Simple, portable options available	More complex, often requires skilled operators	Complex, requires skilled operators
Portability	High (compact, benchtop instruments available)	High (portable versions available)	Low (typically laboratory-based)	Low (laboratory-based)
Analysis Time	Fast (a few minutes per sample)	Fast (a few minutes per sample)	Moderate (longer for high precision)	Slow (preparation and analysis can take hours)
Suitability for Trace Elements	Excellent for trace analysis in environmental, biological, and industrial samples	Moderate, better for higher concentrations	Good for high concentrations but limited for trace elements	Excellent for ultra-trace analysis
Multi-Element Analysis	Yes (simultaneous detection of multiple elements)	Yes (simultaneous detection)	Yes (simultaneous detection with high precision)	Yes (simultaneous detection, including isotopic analysis)
Vacuum/Atmosphere Requirements	Often requires vacuum or helium for light elements	Often requires vacuum or helium for light elements	Requires vacuum or helium	No vacuum needed (operates in solution)
Limitations	Not suitable for very light elements (e.g., H, He, Li, Be)	Lower sensitivity for trace analysis	Bulky and expensive	Requires extensive preparation and has higher operational costs
